# MYC-Mediated Ribosomal Gene Expression Sensitizes Enzalutamide-resistant Prostate Cancer Cells to EP300/CREBBP Inhibitors

**DOI:** 10.1016/j.ajpath.2021.02.017

**Published:** 2021-03-08

**Authors:** Tobias Furlan, Alexander Kirchmair, Natalie Sampson, Martin Puhr, Martina Gruber, Zlatko Trajanoski, Frédéric R. Santer, Walther Parson, Florian Handle, Zoran Culig

**Affiliations:** ∗ Department of Urology, Medical University of Innsbruck, Innsbruck, Austria; † Institute of Bioinformatics, Medical University of Innsbruck, Innsbruck, Austria; ‡ Institute of Legal Medicine, Medical University of Innsbruck, Innsbruck, Austria; § Forensic Science Program, The Pennsylvania State University, University Park, Pennsylvania

## Abstract

Patients with advanced prostate cancer are frequently treated with the antiandrogen enzalutamide. However, resistance eventually develops in virtually all patients, and various mechanisms have been associated with this process. The histone acetyltransferases EP300 and CREBBP are involved in regulation of cellular events in advanced prostate cancer. This study investigated the role of EP300/CREBBP inhibitors in enzalutamide-resistant prostate cancer. EP300/CREBBP inhibitors led to the same inhibition of androgen receptor activity in enzalutamide-resistant and -sensitive cells. However, enzalutamide-resistant cells were more sensitive to these inhibitors in viability assays. As indicated by the RNA-sequencing–based pathway analysis, genes related to the ribosome and MYC activity were significantly altered upon EP300/CREBBP inhibitor treatment. EP300/CREBBP inhibitors led to the down-regulation of ribosomal proteins RPL36 and RPL29. High-level ribosomal proteins amplifications and MYC amplifications were observed in castration-resistant prostate cancer samples of the publicly available Stand Up to Cancer data set. An inhibitor of RNA polymerase I–mediated transcription was used to evaluate the functional implications of these findings. The enzalutamide-resistant cell lines were more sensitive to this treatment. In addition, the migration rate of enzalutamide-resistant cells was strongly inhibited by this treatment. Taken together, the current data show that EP300/CREBBP inhibitors affect the MYC/ribosomal protein axis in enzalutamide-resistant cells and may have promising therapeutic implications.

Prostate cancer (PCa) is one of the most commonly diagnosed types of cancer, especially in industrialized nations. Therapy of localized PCa is mostly curative. For recurrent PCa, androgen deprivation therapy via chemical or surgical castration remains the current standard-of-care treatment.^1^ Although the therapy is initially effective, the development of castration-resistant PCa is nearly always inevitable. In this case, blockade of the androgen receptor (AR) is used, commonly with the antiandrogen enzalutamide. Enzalutamide not only inhibits ligand binding to the AR, but also reduces the nuclear translocation as well as DNA binding of the AR.^2^ Nevertheless, similar to androgen deprivation therapy, enzalutamide is only effective for a short time. Subsequent therapeutic options for enzalutamide-resistant PCa are limited.

Several molecular mechanisms leading to enzalutamide resistance have already been described, including elevated activation of the AR either through overexpression or mutation, expression of AR splice variants, amplification of AR co-activators, enhanced functional activity of glucocorticoid receptor, or MYC overexpression.^3^ The histone acetyl transferase (HAT) EP300 (P300) and its paralogue CREBBP (CBP) are involved in several signaling pathways associated with therapy resistance.^4^

EP300 and CREBBP are multifunctional proteins containing a highly conserved core region that consists of a HAT domain and a bromodomain (BD). The HAT domain catalyzes the transfer of acetyl groups to lysine residues in histones, thereby relaxing the chromatin. However, many other proteins, including EP300 and CREBBP, can be acetylated.^5^ The BD can enhance the efficacy of the HAT domain by binding to acetylated lysines, thus enabling acetylation of several lysines of a target protein.^6^^,^^7^ EP300 and CREBBP are well-known AR coactivators that also acetylate the AR, thus enhancing its activity.^8^ Furthermore, EP300 and CREBBP have been attributed to regulating MYC signaling either via direct interaction or modulation of histones.^9^ The importance of EP300/CREBBP in therapy resistance is further underscored by the observation that they exert oncogenic functions in PCa.^10^ These two coactivators are up-regulated during androgen ablation and implicated in nonsteroidal AR activation. Furthermore, EP300 is associated with regulating cancer hallmarks, including proliferation, apoptosis, migration, and invasion.^4^^,^^11^^,^^12^ EP300 was recently identified as a valid target in a chemotherapeutic setting.^13^ Small molecular inhibitors targeting EP300, its paralogue CREBBP, or related proteins of the bromodomain family have consistently shown promising results in leukemia, colorectal cancer, melanoma, and PCa.^14^, ^15^, ^16^

Based on the involvement of EP300/CREBBP in multiple pathways associated with castration resistance, we hypothesized that targeting the HAT or BD domains of EP300 and CREBBP may regulate cellular processes in enzalutamide-resistant PCa cells. The goal of the current study, therefore, was to investigate the potential of EP300/CREBBP inhibitors in enzalutamide-sensitive and -resistant PCa models and characterize the cellular response. Enzalutamide-resistant cells were highly sensitive to EP300/CREBBP inhibitors. Intriguingly, EP300/CREBBP inhibitors not only antagonized AR function but also down-regulated the expression of ribosomal proteins. Consistently, ribosomal proteins were found to be overexpressed in enzalutamide-resistant cell models as well as in a subgroup of castration-resistant PCa patient samples.

## Materials and Methods

### Cell Culture

LNCaP (Table 1) and PC3 cells were purchased from ATCC (Gaithersburg, MD), and DuCaP cells (Table 1) were a gift from Dr. J.A. Schalken, Nijmegen, the Netherlands.^17^, ^18^, ^19^, ^20^ DuCaP cells have previously been established by xenografting followed by plating of metastatic tissue from the dura mater of a patient with androgen deprivation therapy-resistant PCa into a SCID mouse.^19^ This cell line shows a near triploid karyotype with complex structural rearrangements, loss of heterozygosity of *TP53*, and a high amplification of wild-type AR gene, as well as expression of AR variant 7, which is in contrast to LNCaP cells.^17^

**Table 1:** Overview of Cell Lines Used

Cell line	LNCaP	DuCaP	Ref.
Status	Androgen sensitive	ADT resistant	17,18
Origin of metastatic tissue	Lymph node	Dura mater	18,19
*TP53*	Wt	Heterozygous loss	17
*TMPRSS2-ERG* fusion	No	Yes	20
*AR*	T877A	Wt, gene amplification	17
AR-V7 expression	No	Yes	17

ADT, androgen deprivation therapy.

Enzalutamide-resistant cells were generated by chronic treatment with increasing concentrations of enzalutamide, as described previously.^21^^,^^22^ Enzalutamide-resistant cells are denoted with the suffix EnzaR. All cells were cultured in RPMI 1640 (PAN-Biotech, Aidenbach, Germany) with 10% fetal calf serum (PAN-Biotech), 1% penicillin/streptomycin (Lonza, Basel, Switzerland), and 1% GlutaMAX (Thermo Fisher Scientific, Waltham, MA). Enzalutamide-resistant cells were additionally supplied with 8 μM (DuCaP EnzaR) and 5 μM (LNCaP EnzaR) enzalutamide. The identity of all cell lines was confirmed by short tandem repeat analysis.

For knockdown of MYC, four different pooled siRNA constructs (ON-TARGETplus Human MYC siRNA SMARTPool, THP, Vienna, Austria) or control (ON-TARGETplus siControl SMARTPool, THP) were used. For transfection, Lipofectamine RNAiMAX (Thermo Fisher Scientific) was used according to the manufacturer’s instructions. After 3 days, cells were lysed in radioimmunoprecipitation assay buffer, and protein was blotted.

### Western Blot

A total of 20 μg protein per sample was loaded onto either 3 to 8% Tris-Acetate for EP300/CREBBP detection or 4 to 12% Bis-Tris NuPAGE protein gels (Thermo Fisher Scientific) for electrophoretic separation. Gels were then blotted onto a 0.2 μm Amersham Protran Nitrocellulose membrane (Sigma, St. Louis, MO). Membranes were stained by using Revert 700 Total Protein Stain (LI-COR Biosciences, Lincoln, NE) to quantify total protein. Membranes were incubated in Starting Blocking Buffer (Thermo Fisher Scientific) for 1 hour at room temperature before overnight incubation with a primary antibody at 4°C. After washing with Tris-buffered saline containing 0.1% Tween-20 (hereafter termed TBST), membranes were incubated with IRDye Goat anti-Rabbit/Mouse IgG Secondary Antibody (LI-COR Biosciences) for 45 minutes. The Odyssey imaging System (LI-COR Biosciences) was used to scan membranes after washing with TBST and the Image Studio software (version 5.2, LI-COR Biosciences) to quantify protein amounts. The same blots were probed repeatedly. The following antibodies were used at the indicated dilutions: CREBBP (7389S, Cell Signaling Technology, Danvers, MA; 1:2000), EP300 (ab10485, Abcam, Cambridge, UK; 1:1000), FKBP5/FKBP51(A301-430A, Bethyl, Montgomery, TX; 1:2000), alpha tubulin (sc-5286, Santa Cruz Laboratories, Dallas, TX; 1:2000), MYC (D84C12, Cell Signaling; 1:1000), RPL29 (AP20452c-ev, ABcepta, San Diego, CA; 1:500), RPL36 (ELA-E-AB-32803 to 60, Elabscience Biotechnology, Houston, TX; 1:500), and glyceraldehyde-3-phosphate dehydrogenase (ABS16, Millipore, Burlington, MA; 1:5000).

### Viability Assay

Parental and enzalutamide-resistant DuCaP and LNCaP cells were seeded in a 384-well plate (Corning, Corning, NY) in triplicate at 200 and 2000 cells per well, respectively, and treated with different concentrations of the inhibitors C646 (Sigma), I-CBP112 (Tocris, Bristol, UK), CPI637 (MedChem Express, Monmouth Junction, NJ), CX-5461 (MedChem Express), or the solvent dimethyl sulfoxide (Sigma). RealTime-Glo MT Cell viability assay (Promega, Madison, WI) was used as described by the manufacturer. The Cytation5 (BioTek, Winooski, VT) plate reader equipped with a carbon dioxide incubation chamber and heated to 37°C was used to quantify viability via luminescence over 72 hours. Dose–response curves were generated with GraphPad Prism 8 (GraphPad Software Inc., La Jolla, CA) and 50% inhibitory concentrations were compared by using the sum-of-square-F-test.

### Quantitative Real-Time PCR

Quantitative real-time PCR was performed as previously described.^23^ Total RNA was isolated by using the EXTRACTME RNA Isolation kit (Blirt, Zgierz, Poland) according to the manufacturer’s instructions. LUNAScript kit (New England Biolabs, Ipswich, MA) was used to transcribe 250 ng RNA into cDNA. For quantitative real-time PCR, cDNA was mixed with LUNA Universal Probe RT-qPCR Master Mix (New England Biolabs) and assessed in duplicate on a 7500 Fast Real-Time PCR System (Applied Biosystems, Foster City, CA). Geometric mean of the CT values of reference genes HMBS, HPRT1, and TBP were used for normalization. FKBP5, PSA, and TMPRSS2 were used as targets (Table 2).

**Table 2:** Primer Pairs Used for RT-qPCR

Genes	Gene	Sequence/Catalog no.	Manufacturer
Housekeeping genes	*TBP*	Forward: 5′-CACGAACCACGGCACTGATT-3′	Microsynth AG
		Reverse: 5′-TTTTCTTGCTGCCAGTCTGGAC-3′	
		Probe: 5′- TCTTCACTCTTGGCTCCTGTGCACA-3′-TAMRA	
	*HPRT*	Forward: 5′- GCTTTCCTTGGTCAGGCAGTA-3′	
		Reverse: 5′-GTCTGGCTTATATCCAACACTTCGT-3′	
		Probe: 5′-CAAGGTCGCAAGCTTGCTGGTGAAAAGGA-3′-TAMRA	
	*HMBS*	TaqMan Gene Expression Assay: Hs00609297_m1	Thermo Fisher Scientific
Target genes	*FKBP5*	TaqMan Gene Expression Assay: Hs01561006_m1	Thermo Fisher Scientific
	*TMPRSS2*	TaqMan Gene Expression Assay: Hs01120965_m1	Thermo Fisher Scientific
	*PSA*	Forward: 5′-GTCTGCGGCGGTGTTCTG-3′	Microsynth AG
		Reverse: 5′-TGCCGACCCAGCAAGATC-3′	
		Probe: 5′-CACAGCTGCCCACTGCATCAGGA-3′-TAMRA	

RT-qPCR, quantitative real-time PCR.

### AR Reporter Gene Assay

Parental and enzalutamide-resistant DuCaP and LNCaP cells were seeded in 96-well plates at 2 × 10^4^ and 1 × 10^4^ cells per well, respectively. Cells were transfected by using x-tremeGENE HP (Roche, Basel, Switzerland) with 50 ng pGL4.53 (Promega) encoding firefly luciferase under the control of a constitutive promoter (PGK) for normalization and 50 ng of pGL4.70 (Promega) encoding NanoLuc under the control of a TATA-box preceded by two androgen response elements. After 5 days, cells were treated with inhibitors or R1881. AR activity was measured after 24 hours by using the Nano-Glo Dual-Luciferase Reporter Assay System (Promega) on a Cytation5 microplate reader (BioTek).

### RNA-Sequencing

Parental and enzalutamide-resistant DuCaP and LNCaP cells were seeded in 6-well plates at 8 × 10^5^ and 6 × 10^5^ cells per well. The following day, DuCaP cells were treated with 8 μM enzalutamide, 10 μM C646, 10 μM I-CBP112, or dimethyl sulfoxide equivalent for 24 hours in triplicate. Total RNA was extracted by using the EXTRACTME RNA Isolation kit (Blirt) according to the manufacturer’s instructions. Quality control by Bioanalyzer, poly(A) enrichment, cDNA synthesis, library preparation, Illumina sequencing, and trimming were performed at Microsynth AG (Balgach, Switzerland). One replicate of DuCaP cells was removed for quality analysis. Alignment, counting tables, and differential gene expression analysis were performed by using the online platform *usegalaxy.org* (last accessed June 2020).^24^ Sequences were aligned to HG19 by using HiSat2 version 2.1.0.^25^ HTSeq version 0.61 was used to generate count tables.^26^ Differential gene expression analysis was performed by using DESeq2 version 2.11.^27^ ClusterProfiler version 3.16.0 was used to screen up- or down-regulated genes for overrepresented Gene Ontology version 3.11.4 annotations from Cellular Component.^28^^,^^29^ Up- and down-regulation of gene signatures in DESeq2 data was quantified by using GSEA (Broad Institute, Cambridge, MA).^30^ All tools used standard settings. Data are deposited in Gene Expression Omnibus (*www.ncbi.nlm.nih.gov/geo/GSE163240*; accession number GSE163240).

### Bioinformatics/Patient Data

The mRNA expression (FPKM, polyA) and copy number aberration data for the Stand Up To Cancer data set were downloaded from cBioPortal (dataset version from February 13, 2020). We did not observe any difference compared with the data set version created in July 2020.^31^ Gene set activity was calculated with Gene Set Variation Analysis version 1.36.2 in R statistical software version 4.0.1 (R Foundation for Statistical Computing, Vienna, Austria). The r score and *P* value were calculated by using Pearson correlation. The heatmaps were generated with ComplexHeatmap (version 2.4.3).

### Trans-Well Migration

Parental LNCaP and LNCaP EnzaR cells were treated for 24 hours as described. Cells were harvested and 1 × 10^5^ cells per well were re-treated in serum-free medium and seeded in duplicate into 24-well FluoroBlok inserts with an 8 μm pore size (Corning). Growth medium containing 10% fetal calf serum was used as a chemoattractant in the lower chamber. After 48 hours, cells were stained for 1 hour with 4 μmol/L Calcein AM (Sigma) and dissolved in Hanks’ Balanced Salt Solution (Lonza) with 0.1% bovine serum albumin (Sigma). Staining solution was removed and Hanks’ Balanced Salt Solution with 0.1% bovine serum albumin added. Calcein AM staining was quantified on a Cytation5 microplate reader (BioTek) and representative images taken on a JuLI smart fluorescent cell analyzer (Science Services, Munich, Germany). The percentage of migrated cells was calculated relative to the total number of cells seeded in parallel without inserts but with the same treatment to account for reduced cell number in CX-5461–treated wells.

### Statistical Analysis

For statistical comparison of independent replicates from Western blot, reporter gene assay, quantitative real-time PCR, and Boyden-chamber assay replicates were normalized to the average signal intensity. One-way or two-way analysis of variance with Dunnett’s or Tukey’s multiple comparisons test was used unless stated otherwise. Significant *P* values are noted in the figures as ∗*P* < 0.05, **P < 0.01, and ***P < 0.001.

## Results

### Enzalutamide-resistant Cells Are More Sensitive to EP300/CREBBP Inhibitors than Their Parental Counterparts

**Figure 1:**
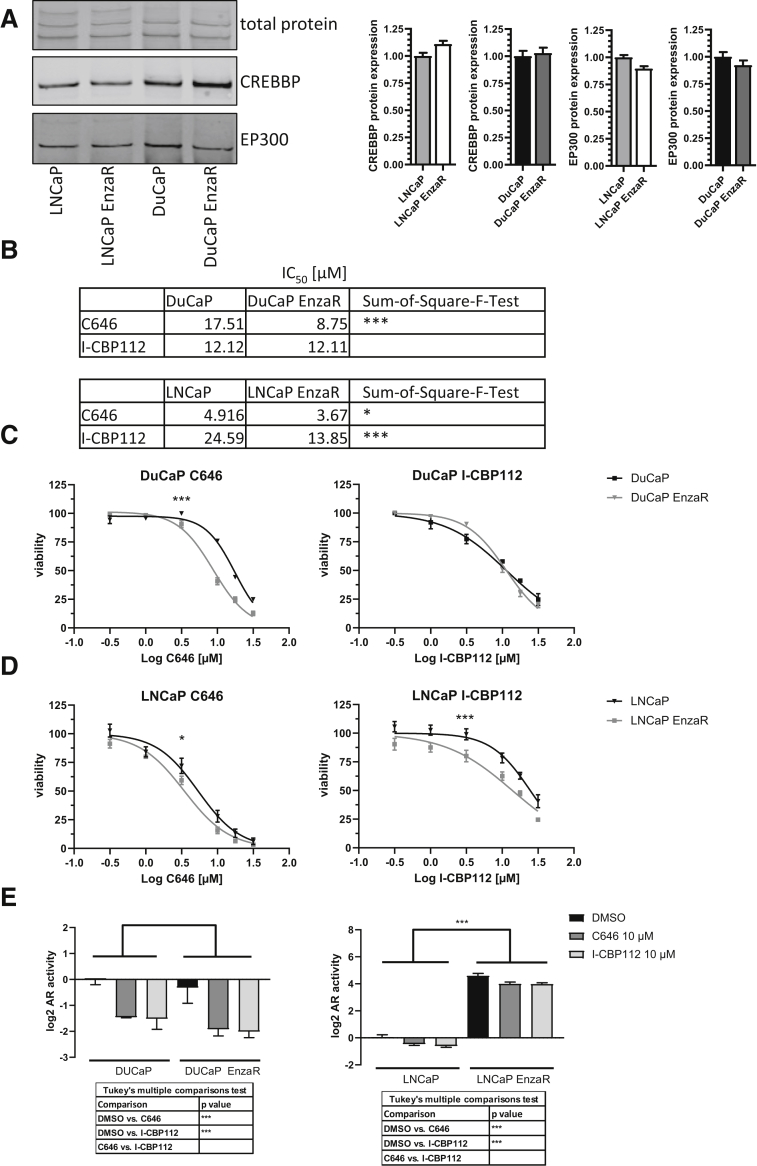
EP300/CREBBP inhibitors in prostate cancer models. **A:** Representative Western blot of DuCaP and LNCaP parental and enzalutamide-resistant cell lysates probed with anti-EP300 and anti-CREBBP antibodies and quantification relative to parental cells (*n* = 3). **B:** Fifty percent inhibitory concentration (IC_50_) of EP300/CREBBP inhibitors C646 and I-CBP112. Corresponding dose–response curve for DuCaP (**C**) and LNCaP (**D**) parental and enzalutamide-resistant cell lines 72 hours after treatment with the indicated inhibitors based on a viability assay (*n* = 3). **E:** Dual luciferase reporter assay for parental and resistant DuCaP and LNCaP cell lines treated with 10 μM of the respective inhibitor over 24 hours (*n* = 4). Numerical data were analyzed by using two-way analysis of variance. Bars indicate SEM. *P* values were calculated by using extra-sum-of-square-F-test for IC_50_. ∗*P* < 0.05, ∗∗∗*P* < 0.001. AR, androgen receptor; DMSO, dimethyl sulfoxide.

To investigate the therapeutic potential of EP300/CREBBP inhibition in the setting of enzalutamide resistance, previously established enzalutamide-resistant DuCaP and LNCaP cell lines (henceforth denoted as DuCaP EnzaR and LNCaP EnzaR, respectively) were used. Protein expression of EP300/CREBBP was clearly detectable, with no significant differences between enzalutamide-sensitive or -resistant cell lines (Figure 1A). Therefore, the growth inhibitory effect of two compounds directed against different domains of EP300 and CREBBP in dose–response proliferation assays were tested. C646 is an inhibitor of the HAT domain, and I-CBP112 targets the BD.^32^^,^^33^ Specific inhibitors of either EP300 or CREBBP are not available due to high sequence similarities.

Viability was strongly reduced in all cell lines upon treatment with the EP300/CREBBP inhibitors, as determined by a real-time cell viability assay (Figure 1B). Interestingly, both enzalutamide-resistant cell lines were more sensitive to C646 inhibitor treatment compared with their nonresistant parental counterparts (Figure 1, C and D). After treatment with the BD inhibitors, only the LNCaP EnzaR cells exhibited a significantly greater sensitivity, compared with parental LNCaP cells (Figure 1, C and D). Because EP300/CREBBP are important AR co-factors, whether AR activity contributed to the higher sensitivity of enzalutamide-resistant cells might be due to effects on AR activity was investigated. To this end, first the basal AR activity and androgen dependency in the cell lines was measured using a dual luciferase AR reporter gene assay (Supplemental Figure S1A). Interestingly, LNCaP EnzaR cells exhibited a strong up-regulation of AR activity compared with that of the parental cells, which was independent of R1881 treatment. In contrast, DuCaP EnzaR cells showed the same response as the parental cells, which suggests that the two enzalutamide-resistant cell lines have different mechanisms of resistance. Importantly, AR activity was reduced upon inhibitor treatment in all cell lines, with little differences between the inhibitors (Figure 1E).These findings were further validated by assessment of AR target gene expression, including KLK3 (PSA), FKBP5, and TMPRSS2, which was also down-regulated (Supplemental Figure S1B). Similar results were also observed at the protein level for FKBP5 (Supplemental Figure S1C). Of note, the EP300/CREBBP inhibitors led to the same relative AR activity reduction in enzalutamide-sensitive and enzalutamide-resistant cell lines. However, the remaining AR activity after inhibitor treatment was still more than 10 times higher in LNCaP EnzaR cells compared with untreated parental cells, which suggests that other pathways are responsible for the observed growth inhibition.

### Regulation of Ribosomal Proteins in Enzalutamide-resistant Cells as Indicated by RNA-Sequencing

**Figure 2:**
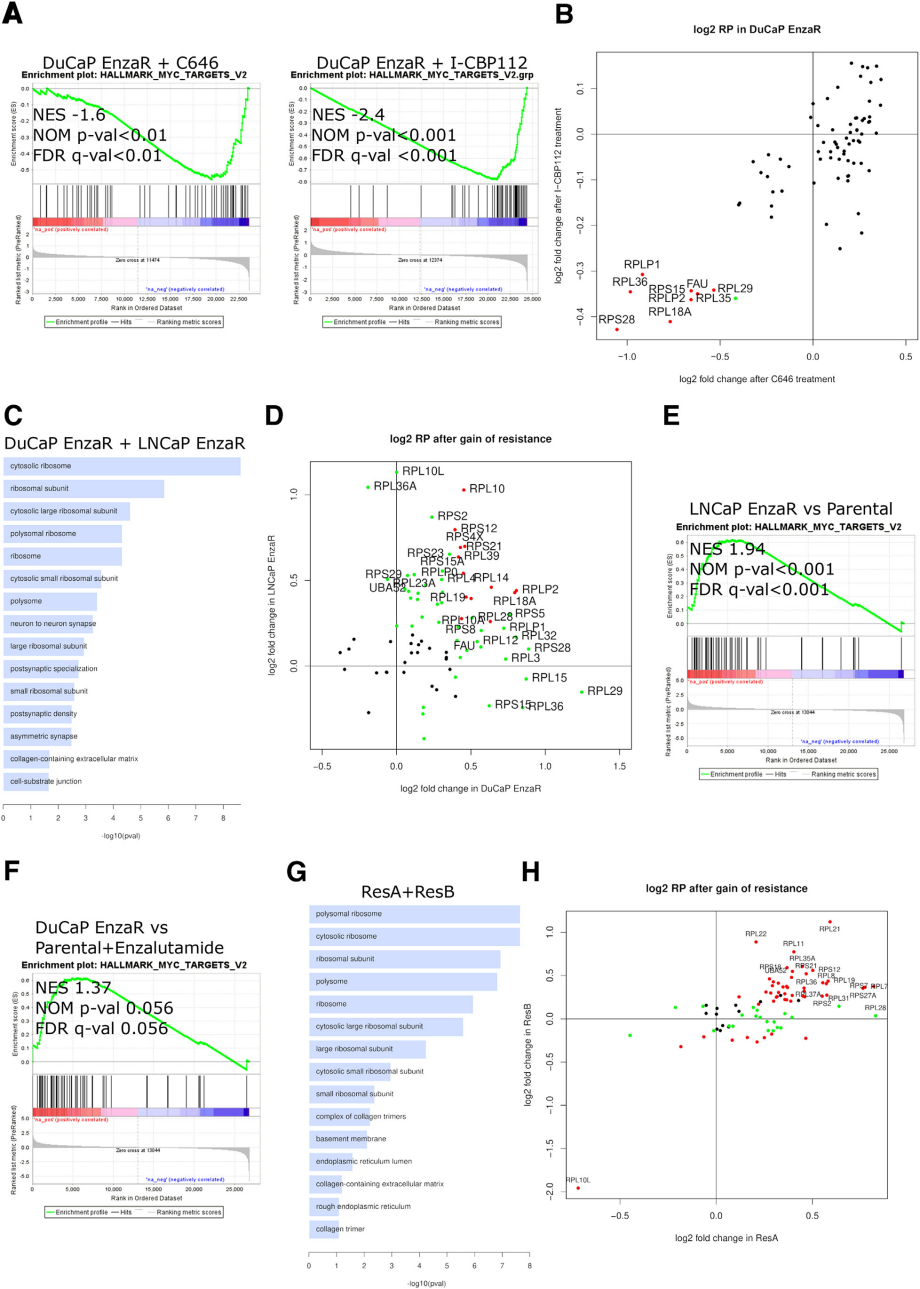
RNA-sequencing of inhibitor treated cells and after development of resistance. For sequencing, parental and enzalutamide-resistant DuCaP (*n* = 2) and LNCaP (*n* = 3) cells were treated with 8 μM enzalutamide, 10 μM C646, 10 μM I-CBP112, or dimethyl sulfoxide equivalent for 24 hours. **A:** GSEA with MYC V2 (MSigDB version 5.0) signature of differentially regulated genes of DuCaP EnzaR after C646 or I-CBP112 treatment compared with vehicle-treated DuCaP Enza. **B:** Expression levels of all ribosomal proteins in DuCaP EnzaR treated with C646 or I-CBP112. Genes significantly regulated in one cell line or both cell lines are denoted in green and red, respectively. Non-significantly regulated genes are colored in black. **C:** Gene Ontology analysis of overlapping genes up-regulated in resistant DuCaP and LNCaP compared with respective parental cells for cellular components. **D:** Expression levels of all ribosomal proteins in DuCaP EnzaR and LNCaP Enza. Significantly regulated genes in one cell line are green and in both are red; not significantly regulated genes are black. GSEA with MYC V2 (MSigDB version 5.0) signature of differentially regulated genes of LNCaP EnzaR compared with parental cells (**E**) and DuCaP EnzaR versus parental cells (**F**) under enzalutamide treatment. **G:** Gene Ontology analysis of overlapping genes up-regulated in ResA and ResB compared with parental cells for cellular components. **H:** Expression levels of all ribosomal proteins in ResA and ResB. Significantly regulated genes in one cell line are green and in both are red; not significantly regulated genes are black. FDR, false discovery rate; NES, normalized enrichment score; NOM, nominal.

To identify the down-stream effects of the inhibitors, RNA-sequencing was performed in DuCaP EnzaR cells treated with 10 μM of C646 or I-CBP112 for 24 hours. GSEA pathway analysis indicated a significant reduction of the MYC activity signature after treatment with either inhibitor (Figure 2A, Supplemental Tables S1–S4). Moreover, Gene Ontology analysis of genes commonly down-regulated in both cell lines (Supplemental Tables S5–S7) revealed structural constituents of the ribosome as an overrepresented term. Further analysis revealed that a subset of genes encoding ribosomal proteins was down-regulated after treatment with either inhibitor (Figure 2B). This finding is in line with previous publications that show MYC-dependent regulation of ribosomal proteins.^34^

RNA sequencing was used to examine why EP300/CREBBP inhibitors affect enzalutamide-resistant DuCaP and LNCaP cells more than enzalutamide-sensitive parental cells. Consistent with findings presented in the previous paragraph, Gene Ontology analysis of genes commonly up-regulated in DuCaP EnzaR and LNCaP EnzaR cells compared with untreated parental cell lines revealed an overrepresentation of genes related to the ribosome and collagen-containing extracellular matrix (Figure 2C, Supplemental Tables S8–S10). A more detailed analysis revealed that the majority of genes encoding for ribosomal proteins were up-regulated in at least one cell line, although there was only a partial overlap between the two cell lines (Figure 2D). Consistently, GSEA pathway analysis showed that the MYC activity signature was strongly enriched in LNCaP EnzaR cells (Figure 2E, Supplemental Tables S11 and S12) and moderately activated in DuCaP EnzaR cells, as shown by comparison with enzalutamide-treated parental DuCaP cells (Figure 2F).

To further confirm these results, a second publicly available RNA-sequencing data set with two long-term antiandrogen- treated cell lines (ResA and ResB) that are highly resistant to enzalutamide was analyzed.^35^ Although these cells are also derived from LNCaP, they have low basal AR activity (AR indifferent) as opposed to LNCaP EnzaR cells (Figure 1E). Importantly, an overrepresentation of up-regulated genes encoding ribosomal genes in ResA and ResB cells compared with normal LNCaP cells was observed (Figure 2, G and H).

### Ribosomal Proteins Are Up-Regulated in Enzalutamide-resistant Cells and Downregulated after EP300/CREBBP Inhibitor Treatment

**Figure 3:**
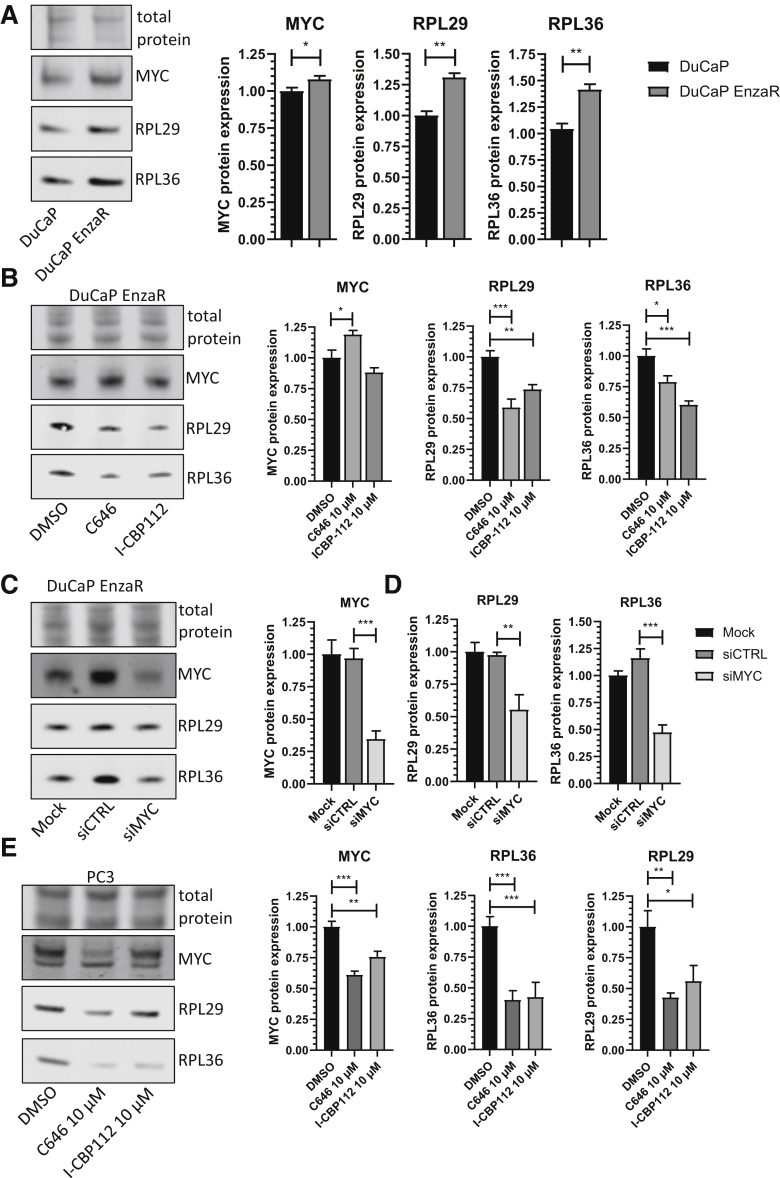
Validation of MYC, RPL29, and RPL36 expression. **A:** Representative Western blots and quantification of parental and enzalutamide-resistant DuCaP cells probed for MYC, RPL29, and RPL36 (*n* = 6). **B:** Representative Western blots and quantification for DuCaP EnzaR cells treated with 10 μM C646, 10 μM I-CBP112, or dimethyl sulfoxide (DMSO) for 48 hours, probed for MYC, RPL29, and RPL36 (*n* = 6). **C:** Pooled siRNAs either targeting MYC (siMYC) or nontargeting control (siCTRL) were used to establish and validate MYC knockdown together with mock treatment (Mock). Knockdown efficiency was validated by Western blot with an antibody for MYC. **D:** The blots were probed with antibodies specific for RPL36 and RPL29. For quantification, MYC, RPL36, and RPL29 were normalized to mock-treated cells (*n* = 3). **E:** PC3 cells were treated for 48 hours with 10 μM C646 or 10 μM I-CBP112 (*n* = 3). Blots were probed with MYC, RPL36, and RPL29 specific antibodies. Numerical data were analyzed via one-way analysis of variance. ∗*P* < 0.05, ∗∗*P* < 0.01, ∗∗∗*P* < 0.001.

The protein expression of MYC and two of the most strongly regulated ribosomal proteins in DuCaP cells was measured after gain of resistance or treatment with inhibitors, *RPL36* and *RPL29*. In line with the RNA-sequencing data, an increased protein expression of RPL36 and RPL29 was observed in DuCaP EnzaR cells (Figure 3A), which could be reversed by treatment with the EP300/CREBBP inhibitors (Figure 3B). Of note, the inhibitors did not reduce MYC protein expression, suggesting a more complex influence on MYC activity.

siRNA knockdown was performed to verify whether MYC knock-down was sufficient to regulate the expression of ribosomal proteins (Figure 3C). Consequently, MYC knock-down was found to be sufficient to down-regulate protein expression of RPL36 and RPL29 (Figure 3D). AR-negative PC3 cells were used to determine whether EP300/CREBBP inhibitors could also reduce ribosomal protein expression, independently of AR. Indeed, both C646 and I-CBP112 were able to reduce RPL36 and RPL29 expression (Figure 3E). MYC was also down-regulated in PC3 cells upon inhibitor treatment.

### Expression of Ribosomal Proteins in Patient Samples

**Figure 4:**
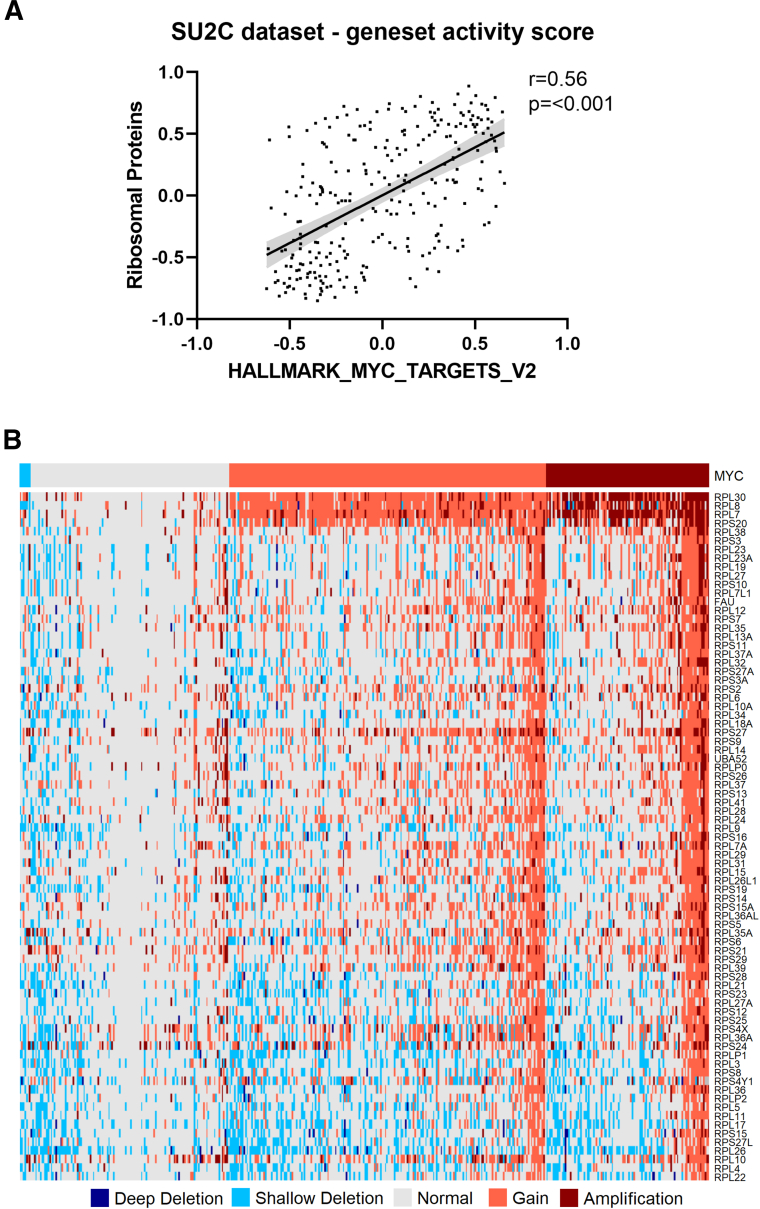
MYC and ribosomal proteins in samples of patients with metastatic prostate cancer. **A:** Correlation of MYC target and ribosomal protein gene-set variation analysis scores from the publicly available Stand Up To Cancer data sets for metastatic prostate cancer. Graph is shown with linear regression (black line) and 95% confidence intervals (gray shaded area). The r score and *P* value were calculated by using Pearson correlation. **B:** Correlation of copy number aberrations in genes for ribosomal proteins and MYC. Copy number aberrations are separated as high-level amplification (2), low-level gain (1), diploid (0), shallow loss (−1), and deep loss (−2).

To confirm the clinical relevance of the findings in patients with PCa, MYC activity and the mRNA expression of ribosomal proteins were investigated in primary and metastatic castration-resistant PCa samples from the Stand Up To Cancer data set. In addition to that in cell lines, mRNA of ribosomal proteins and MYC were also analyzed in patient data. Indeed, a statistically significant correlation was observed between MYC gene set activity and the mRNA expression of ribosomal proteins (Figure 4A). However, MYC expression was not the only factor that correlated with overexpression of ribosomal proteins. Copy number aberrations were common for ribosomal proteins (Figure 4B). Interestingly, high-level amplifications of *RPL7, RPL30, RPL8,* and *RPS20* were common in up to 27% of patients. All three amplifications often co-occurred with amplifications of *MYC*, as shown by clustering (Figure 4B). This is not surprising because of their proximity on chromosome 8. Amplifications of parts of chromosome 8 are common.^36^ However, ribosomal proteins not located on chromosome 8, including *RPS27* and *RPS24*, were also highly amplified in 10% of patients.

### Inhibition of Ribosomal Proteins Affects Cellular Viability and Migration in PCa

**Figure 5:**
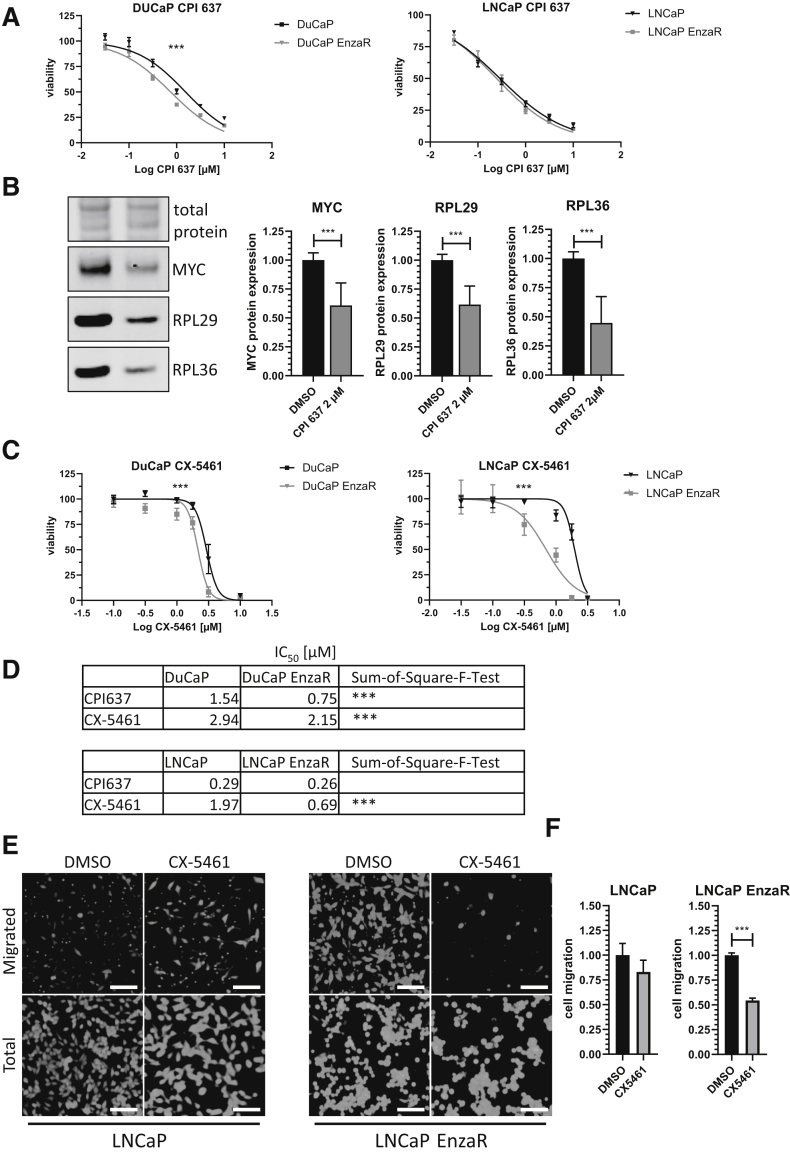
Next-generation bromodomain inhibitor and ribosomal neogenesis inhibitor in prostate cancer cell lines. **A:** Dose–response curve for parental and resistant DuCaP and LNCaP cells after 72 hours of CPI637 treatment. **B:** Representative Western blots for DuCaP EnzaR cells treated with 2 μM CPI637 for 48 hours, probed for MYC, RPL29, and RPL36 (*n* = 3). **C:** Dose–response curve for parental and resistant DuCaP and LNCaP cells after 72 hours of CX-5461 treatment. **D:** Fifty percent inhibitory concentration (IC_50_) of CPI637 and CX-5461 for DuCaP and LNCaP parental and enzalutamide-resistant cell lines 72 hours after treatment based on a viability assay (*n* = 3). *P* value was calculated using extra-sum-of-square-F-test for IC_50_. **E:** Boyden chamber assay with parental and LNCaP EnzaR cells, with or without 2 μM CX-5461 for 72 hours. Cells were stained with Calcein AM. **Upper row** shows migrated cells, and **lower row** shows total cells. (**F**) Graph shows relative fluorescence intensity to untreated cells, normalized to wells with same treatment but without insert. Numerical data were analyzed via one-way analysis of variance. Bars indicate SEM (*n* = 3) (**A** and **C**). ∗∗∗*P* < 0.001. Scale bars: 100 μm (**E**). DMSO, dimethyl sulfoxide.

Although C646 and I-CBP112 are useful tools for research, they are not well-suited for clinical therapy due to their low half-life and selectivity.^37^^,^^38^ To overcome this problem, CPI637, a newer generation BD inhibitor^39^ was tested (Figure 5, A and B). CPI637 reached a nanomolar 50% inhibitory concentration in both enzalutamide-resistant cell lines tested, and had a higher efficiency in DuCaP EnzaR cells compared with enzalutamide-sensitive parental cells (Figure 5C).

These results show that ribosomal proteins are up-regulated not only in enzalutamide-resistant cells but also in patient samples. To investigate the potential utility of targeting ribosomal proteins, an inhibitor of RNA polymerase I–directed transcription (CX-5461), which is responsible for transcription of rRNA and thereby inhibits ribosome formation, was used. Indeed, enzalutamide-resistant cells were significantly more susceptible to CX-5461 than their enzalutamide-sensitive parental counterparts (Figure 5D).

Expression of ribosomal proteins correlates with increased migration and invasion potential.^40^^,^^41^ To test whether and to what extent ribosomal proteins regulate migration in our cell models, a migration assay was performed with parental LNCaP and LNCaP EnzaR cell lines. DuCaP cells are not suited for such assays due to their growth pattern. CX-5461 treatment of enzalutamide-resistant LNCaP cells, but not parental cells, resulted in significantly lower migration (Figure 5, E and F).

## Discussion

EP300/CREBBP function as oncogenes in PCa via potentiation of ligand-independent AR activation, regulation of AR target gene expression in androgen-insensitive cells, and activation of cellular processes such as migration and invasion.^4^^,^^8^^,^^42^ Recently, EP300 was also identified as a target in chemotherapy-resistant PCa cells.^13^ Furthermore, EP300 and CREBBP are up-regulated during androgen ablation.^12^ As with EP300, CREBBP is involved in regulating the antagonist/agonist balance of antiandrogens.^10^ Thus, CREBBP and EP300 may have distinct functions in prostate carcinogenesis, depending on tumor stage and cell model used.

Several different mechanisms of enzalutamide resistance, including mutations and amplification of the *AR*,^43^ enhanced activity of co-activators such as GREB1,^44^ or increased activity of the glucocorticoid receptor, have been described.^21^^,^^45^^,^^46^ Overexpression of AR, the AR variant 7, and glucocorticoid receptor have been identified in the cell models used in this study.^21^^,^^22^ In addition, the current study shows that MYC signaling is elevated in enzalutamide-resistant cell models. The highest expression of wild-type and variant AR in cellular models developed by our laboratory was seen in DuCaP cells, which were also used in the current study.

Interestingly, lower concentrations of EP300/CREBBP inhibitors were sufficient to decrease viability in several models of enzalutamide-resistant cells compared with their nonresistant parental counterparts. However, as shown in luciferase assays, this decreased viability in response to both inhibitors was not caused by decreased AR activity. Therefore, RNA-sequencing was performed to identify potential molecular basis for reduced viability in cells treated with BD/HAT inhibitors. The RNA-sequencing experiments indicated that enzalutamide resistance was associated with changes in both cell lines. Although modulation of AR signaling differed between DuCaP EnzaR and LNCaP EnzaR cells, overexpression of ribosomal proteins and activation of MYC signaling was a common factor, confirmed by bioinformatics analysis. Although an up-regulation of many ribosomal proteins in DuCaP EnzaR and LNCaP EnzaR cells was observed, approximately one-third of ribosomal proteins showed no altered expression. This finding suggests that the overexpressed ribosomal proteins may not be involved in ribosome formation per se, which would require a homogeneous distribution of ribosomal proteins. Notably, a shift in the transcriptome has been reported for heterogeneous ribosomes.^47^

RPL36 and RPL29, that were overexpressed in enzalutamide-resistant DuCaP cells and down-regulated upon BD/HAT inhibitor treatment, were quantified to validate the RNA-sequencing findings. A potential role of other ribosomal proteins in PCa pathogenesis has previously been reported, including RPL19, whose knockdown in the AR-negative cell line PC3 reduces invasive potential and tumorigenicity.^40^ Signaling pathway analysis of RPL19-depleted cells identified changes in transcription factor networks and cellular adhesion genes. Besides RPL19, the ribosomal proteins 21 and 24 have been proposed as possible PCa biomarkers.^48^ Taken together, previous results and those reported here open the possibility to investigate multiple roles of ribosomal proteins in PCa and their contribution to therapy resistance.

Up-regulation of ribosomal proteins is not a peculiarity of our cell models but was also observed in those reported by Handle et al.^35^ Interestingly, in that study, AR activity was not required for growth of enzalutamide-resistant cells. In the current study, the enzalutamide-resistant cell lines were also more susceptible toward an inhibitor of ribosomal biogenesis, which not only reduced viability but also cell migration. These results indirectly confirm those of Bee et al,^40^ who emphasized the importance of ribosomal proteins for the invasive phenotype. The current results may be particularly interesting in view of those recently published by Ebright at al,^41^ wherein RPL15 overexpression promoted metastatic breast cancer growth and circulating tumor cells from patients with breast cancer displayed ribosome and protein synthesis signatures. Taken together, data from prostate and breast cancer studies indicate that ribosomal proteins may have a key role in the progression of endocrine-related malignancies. Whether specific subgroups of ribosomal proteins have redundant functions in cancer remains to the investigated. The responsiveness of enzalutamide-resistant cells to CX-5461 in viability and migration studies was similar to that observed with EP300/CREBBP inhibitors.

To analyze EP300/CREBBP action in enzalutamide-resistant cells in detail, MYC function was inhibited in the current study via siRNA-mediated knockdown. MYC knockdown led to the down-regulation of several ribosomal proteins. In addition, analysis of patient data confirmed that up-regulation of ribosomal proteins correlated with MYC activity. These results are consistent with several previous reports indicating ribosomal protein regulation by MYC and the established role of MYC as a regulator of ribosome biogenesis, function, and protein synthesis.^49^ Involvement of MYC overexpression as a mechanism for resistance to enzalutamide is reported, in part, in cells that display reduced AR expression in case of enzalutamide resistance.^35^^,^^50^ Interactions between MYC and the AR signaling pathway are bidirectional. Treatment with EP300/CREBBP inhibitors not only reduces AR but also MYC signaling.^51^

Although the current study focused on inhibition of EP300/CREBBP in association with MYC/ribosomal protein regulation, other possible actions of these inhibitors should be mentioned. Recently, Fan et al^52^ reported that enzalutamide-resistant C4-2 cells are more sensitive to either BET or EP300/CREBBP inhibitors than parental cells. They attributed the effect to the destabilization of the histone demethylase JMJD1A, which is acetylated by EP300. Importantly, the current results, which were obtained using models different from those of Fan et al, are in concordance regarding the use of those inhibitors in enzalutamide-resistant PCa.

Studies using EP300/CREBBP inhibitors in a PCa background suggest that suppression of AR signaling is responsible for the observed inhibition of proliferation. Here, inhibition of MYC signaling and subsequent down-regulation of ribosomal proteins were also shown to be involved. This correlates with results showing that the EP300/CREBBP HAT inhibitor C646 is also effective in the AR-negative cell line PC3.^53^ In clinical trials for acute myeloid leukemia, EP300 and CREBBP inhibitors have limited side effects or cytotoxic effects. For the treatment of PCa with BD inhibitors, phase 2a clinical trials show promising results.^54^ In recent years, generations of EP300/CREBBP inhibitors with higher specificity and potency have been developed, suggesting that results from previous clinical trials could be further improved.

In conclusion, our results reveal that targeting EP300/CREBBP in advanced PCa is not limited to AR signaling but also involves the MYC/ribosomal protein axis. Findings reported herein open possibilities to elucidate the role of ribosomal proteins in PCa. Therefore, clinical targeting of ribosomal proteins either through newer generation of EP300/CREBBP inhibitors or directly targeting ribosomes may be a promising strategy to treat advanced PCa in the future.

## Supplemental Data

Supplemental Figure S1 **A:** Dual luciferase reporter assay for parental and resistant DuCaP and LNCaP cell lines treated with 10 nmol/L R1881 or dimethyl sulfoxide (DMSO) over 24 hours (*n* = 4). **B:** Quantitative real-time PCR of parental and enzalutamide-resistant DuCaP treated with DMSO, 10 μM C646, or 10 μM I-CBP112 for 24 hours in duplicates probed with primers specific for FKBP5, PSA, and TMPRSS2. Signals were normalized to the geometric mean of TBP, HPRT1, and HMBS expression (*n* = 3). Data are shown relative to DMSO-treated control. **C:** Representative Western blots for parental and resistant DuCaP and LNCaP cells treated with 10 μM C646, 10 μM I-CBP112, and 8 or 5 μM enzalutamide for 48 hours, probed for FKBP5 (*n* = 6). Numerical data were analyzed via one-way analysis of variance. ∗*P* < 0.05, ∗∗*P* < 0.01, ∗∗∗*P* < 0.001.

## References

[1] Barsouk A., Padala S.A., Vakiti A., Mohammed A., Saginala K., Thandra K.C., Rawla P., Barsouk A. (2020). Epidemiology, staging and management of prostate cancer. Med Sci (Basel).

[2] Tran C., Ouk S., Clegg N.J., Chen Y., Watson P.A., Arora V., Wongvipat J., Smith-Jones P.M., Yoo D., Kwon A., Wasielewska T., Welsbie D., Degui Chen C., Higano C.S., Beer T.M., Hung D.T., Scher H.I., Jung M.E., Sawyers C.L. (2009). Development of a second-generation antiandrogen for treatment of advanced prostate cancer. Science.

[3] Prekovic S., van der Broeck T., Linder S., van Royen M.E., Houtsmuller A.B., Handle F., Joniau S., Zwart W., Claessens F. (2018). Molecular underpinnings of enzalutamide resistance. Endocr Relat Cancer.

[4] Debes J.D., Schmidt L.J., Huang H., Tindall D.J. (2002). p300 Mediates androgen-independent transactivation of the androgen receptor by interleukin 6. Cancer Res.

[5] Dancy B.M., Cole P.A. (2015). Protein lysine acetylation by p300/CBP. Chem Rev.

[6] Chen J., Ghazawi F.M., Li Q. (2010). Interplay of bromodomain and histone acetylation in the regulation of p300-dependent genes. Epigenetics.

[7] Weinert B.T., Narita T., Satpathy S., Srinivasan B., Hansen B.K., Schölz C., Hamilton W.B., Zucconi B.E., Wang W.W., Liu W.R., Brickman J.M., Kesicki E.A., Lai A., Bromberg K.D., Cole P.A., Choudhary C. (2018). Time-resolved analysis reveals rapid dynamics and broad scope of the CBP/p300 acetylome. Cell.

[8] Fu M., Wang C., Reutens A.T., Wang J., Angeletti R.H., Siconolfi-Baez L., Ogryzko V., Avantaggiati M.L., Pestell R.G. (2000). p300 and p300/cAMP-response element-binding protein-associated factor acetylate the androgen receptor at sites governing hormone-dependent transactivation. J Biol Chem.

[9] Vervoorts J., Lüscher-Firzlaff J.M., Rottmann S., Lilischkis R., Walsemann G., Dohmann K., Austen M., Lüscher B. (2003). Stimulation of c-MYC transcriptional activity and acetylation by recruitment of the cofactor CBP. EMBO Rep.

[10] Comuzzi B., Nemes C., Schmidt S., Jasarevic Z., Lodde M., Pycha A., Bartsch G., Offner F., Culig Z., Hobisch A. (2004). The androgen receptor co-activator CBP is up-regulated following androgen withdrawal and is highly expressed in advanced prostate cancer. J Pathol.

[11] Ianculescu I., Wu D.Y., Siegmund K.D., Stallcup M.R. (2012). Selective roles for cAMP response element-binding protein binding protein and p300 protein as coregulators for androgen-regulated gene expression in advanced prostate cancer cells. J Biol Chem.

[12] Heemers H.V., Sebo T.J., Debes J.D., Regan K.M., Raclaw K.A., Murphy L.M., Hobisch A., Culig Z., Tindall D.J. (2007). Androgen deprivation increases p300 expression in prostate cancer cells. Cancer Res.

[13] Gruber M., Ferrone L., Puhr M., Santer F.R., Furlan T., Eder I.E., Sampson N., Schäfer G., Handle F., Culig Z. (2020). p300 is upregulated by docetaxel and is a target in chemoresistant prostate cancer. Endocr Relat Cancer.

[14] Yan G., Eller M.S., Elm C., Larocca C.A., Ryu B., Panova I.P., Dancy B.M., Bowers E.M., Meyers D., Lareau L., Cole P.A., Taverna S.D., Alani R.M. (2013). Selective inhibition of p300 HAT blocks cell cycle progression, induces cellular senescence, and inhibits the DNA damage response in melanoma cells. J Invest Dermatol.

[15] Liu Y., Yang E.J., Shi C., Mou P.K., Zhang B., Wu C., Lyu L., Shim J.S. (2020). Histone acetyltransferase (HAT) P300/CBP inhibitors induce synthetic lethality in PTEN-deficient colorectal cancer cells through destabilizing AKT. Int J Biol Sci.

[16] Giotopoulos G., Chan W.I., Horton S.J., Ruau D., Gallipoli P., Fowler A., Crawley C., Papaemmanuil E., Campbell P.J., Göttgens B., Van Deursen J.M., Cole P.A., Hunthly B.J.P. (2016). The epigenetic regulators CBP and p300 facilitate leukemogenesis and represent therapeutic targets in acute myeloid leukemia. Oncogene.

[17] van Bokhoven A., Varella-Garcia M., Korch C., Johannes W.U., Smith E.E., Miller H.L., Nordeen S.K., Miller G.J., Lucia M.S. (2003). Molecular characterization of human prostate carcinoma cell lines. Prostate.

[18] Horoszewicz J.S., Leong S.S., Kawinski E., Karr J.P., Rosenthal H., Chu T.M., Mirand E.A., Murphy G.P. (1983). LNCaP model of human prostatic carcinoma. Cancer Res.

[19] Lee Y.G., Korenchuk S., Lehr J., Whitney S., Vessela R., Pienta K.J. (2001). Establishment and characterization of a new human prostatic cancer cell line: DuCaP. In Vivo.

[20] Saramäki O.R., Harjula A.E., Martikainen P.M., Vessella R.L., Tammela T.L.J., Visakorpi T. (2008). TMPRSS/ERG fusion identifies a subgroup of prostate cancers with a favorable prognosis. Clin Cancer Res.

[21] Puhr M., Hoefer J., Eigentler A., Ploner C., Handle F., Schaefer G., Kroon J., Leo A., Heidegger I., Eder I., Culig Z., Van der Pluijm G., Klocker H. (2018). The glucocorticoid receptor is a key player for prostate cancer cell survival and a target for improved antiandrogen therapy. Clin Cancer Res.

[22] Hoefer J., Akbor M., Handle F., Ofer P., Puhr M., Parson W., Culig Z., Klocker H., Heidegger I. (2016). Critical role of androgen receptor level in prostate cancer cell resistance to new generation antiandrogen enzalutamide. Oncotarget.

[23] Puhr M., Santer F.R., Neuwirt H., Susani M., Nemeth J.A., Hobisch A., Kenner L., Culig Z. (2009). Down-regulation of suppressor of cytokine signaling-3 causes prostate cancer cell death through activation of the extrinsic and intrinsic apoptosis pathways. Cancer Res.

[24] Afgan E., Baker D., Batut B., van den Beek M., Bouvier D., Cech M., Chilton J., Clements D., Coraor N., Grüning B.A., Guerler A., Hillman-Jackson J., Hiltermann S., Jalili V., Rasche H., Soranzo N., Goecks J., Taylor J., Nekrutenko A., Blankenberg D. (2018). The Galaxy platform for accessible, reproducible and collaborative biomedical analyses: 2018 update. Nucleic Acids Res.

[25] Kim D., Langmead B., Salzberg S.L. (2015). HISAT: a fast spliced aligner with low memory requirements. Nat Meth.

[26] Anders S., Pyl P.T., Huber W. (2015). HTSeq—a Python framework to work with high-throughput sequencing data. Bioinformatics.

[27] Love M.I., Huber W., Anders S. (2014). Moderated estimation of fold change and dispersion for RNA-seq data with DESeq2. Genome Biol.

[28] Ashburner M., Ball C.A., Blake J.A., Botstein D., Butler H., Cherry J.M., Davis A.P., Dolinski K., Dwight S.S., Eppig J.T., Harris M.A., Hill D.P., Issel-Tarver L., Kasarskis A., Lewis S., Matese J.C., Richardson J.E., Ringwald M., Rubin G.M., Sherlock G. (2000). Gene ontology: tool for the unification of biology. The Gene Ontology Consortium. Nat Genet.

[29] Yu G., Wang L.G., Han Y., He Q.Y. (2012). clusterProfiler: an R package for comparing biological themes among gene clusters. OMICS.

[30] Subramanian A., Tamayo P., Mootha V.K., Mukherjee S., Ebert B.L., Gillette M.A., Paulovich A., Pomeroy S.L., Golub T.R., Lander E.S., Mesirov J.P. (2005). Gene set enrichment analysis: a knowledge-based approach for interpreting genome-wide expression profiles. Proc Natl Acad Sci U S A.

[31] Abida W., Cyrta J., Heller G., Prandi D., Amenia J., Coleman I. (2019). Genomic correlates of clinical outcome in advanced prostate cancer. Proc Natl Acad Sci U S A.

[32] Bowers E.M., Yan G., Mukherjee C., Orry A., Wang L., Holbert M.A., Crump N.T., Hazzalin C.A., Lisczak G., Yuan H., Larocca C., Saldanha S.A., Abagyarn R., Sun Y., Meyers D.J., Marmorstein R., Mahadevan L.C., Alani R.M., Cole P.A. (2010). Virtual ligand screening of the p300/CBP histone acetyltransferase: identification of a selective small molecule inhibitor. Chem Biol.

[33] Picaud S., Fedorov O., Thanasopoulou A., Leonards K., Jones K., Meier J. (2015). Generation of a selective small molecule inhibitor of the CBP/p300 bromodomain for leukemia therapy. Cancer Res.

[34] Ji H., Wu G., Zhan X., Nolan A., Koh C., De Marzo A., Mai Doan H., Fan J., Cheadle C., Fallahi M., Cleveland J.L., Dang C.V., Zeller K.I. (2011). Cell-type independent MYC target genes reveal a primordial signature involved in biomass accumulation. PLoS One.

[35] Handle F., Prekovic S., Helsen C., Van den Broeck T., Smeets E., Moris L., Eerlings R., El Kharraz S., Urbanucci A., Mills I.G., Joniau S., Attard, Claessens F. (2019). Drivers of AR indifferent anti-androgen resistance in prostate cancer cells. Sci Rep.

[36] El Gammal A.T., Brüchmann M., Zustin J., Isbarn H., Hellwinkel O.J.C., Köllermann J., Sauter G., Simon R., Wilczak W., Schwarz J., Bokemeyer C., Brümmendorf T.H., Izbicki J.R., Yekebas E., Fisch M., Huland H., Graefen M., Schlomm T. (2010). Chromosome 8p deletions and 8q gains are associated with tumor progression and poor prognosis in prostate cancer. Clin Cancer Res.

[37] Cermakova K., Hodges H.C. (2018). Next-generation drugs and probes for chromatin biology: from targeted protein degradation to phase separation. Molecules.

[38] Shortt J., Ott C.J., Johnstone R.W., Bradner J.E. (2017). A chemical probe toolbox for dissecting the cancer epigenome. Nat Rev Cancer.

[39] Taylor A.M., Côté A., Hewitt M.C., Pastor R., Leblanc Y., Nasveschuk C.G. (2016). Fragment-based discovery of a selective and cell-active benzodiazepinone CBP/EP300 bromodomain inhibitor (CPI-637). ACS Med Chem Lett.

[40] Bee A., Brewer D., Beesley C., Dodson A., Forootan S., Dickinson T., Gerard P., Lane B., Yao S., Cooper C.S., Djamgoz M.B.A., Gosden C.M., Ke Y., Foster C.S. (2011). siRNA knockdown of ribosomal protein gene RPL19 abrogates the aggressive phenotype of human prostate cancer. PLoS One.

[41] Ebright R.Y., Lee S., Wittner B.S., Niederhoffer K.L., Nicholson B.T., Bardia A., Truesdell S., Wiley D.F., Wesley B., Li S., Mai A., Aceto N., Vincent-Jordan N., Szabolcs A., Chirn B., Kreuzer J., Comaills V., Kalinich M., Haas W., Ting D.T., Toner M., Vasudevan S., Haber D.A., Maheswaran S., Micalizzi D.S. (2020). Deregulation of ribosomal protein expression and translation promotes breast cancer metastasis. Science.

[42] Shin S.H., Lee G.Y., Lee M., Kang J., Shin H.W., Chun Y.S., Park J.W. (2018). Aberrant expression of CITED2 promotes prostate cancer metastasis by activating the nucleolin-AKT pathway. Nat Comm.

[43] Blatt E.B., Raj G.V. (2019). Molecular mechanisms of enzalutamide resistance in prostate cancer. Cancer Drug Resist.

[44] Lee E., Wongvipat J., Choi D., Wang P., Lee Y.S., Zheng D., Watson P.A., Gopalan A., Sawyers C.L. (2019). *GREB1* amplifies androgen receptor output in human prostate cancer and contributes to antiandrogen resistance. Elife.

[45] Shah N., Wang P., Wongvipat J., Karthaus W.R., Abida W., Armenia J., Rockowitz S., Drier Y., Bernstein B.E., Long H.W., Mreedman M.L., Arora V.K., Zheng D., Sawyers C.L. (2017). Regulation of the glucocorticoid receptor via a BET-dependent enhancer drives antiandrogen resistance in prostate cancer. Elife.

[46] Arora V.K., Schenkein E., Murali R., Subudhi S.K., Wongvipat J., Balbas M.D., Shah N., Cai L., Efstathiou E., Logothetis C., Zheng D., Sawyers C.L. (2013). Glucocorticoid receptor confers resistance to antiandrogens by bypassing androgen receptor blockade. Cell.

[47] Shi Z., Fujii K., Kovary K.M., Genuth N.R., Röst H.L., Teruel M.N., Barna M. (2017). Heterogeneous ribosomes preferentially translate distinct subpools of mRNAs genome-wide. Mol Cell.

[48] Arthurs C., Murtaza B.N., Thomson C., Dickens K., Henrique R., Patel H.R.H., Beltran M., Millar M., Thrasivolou C., Ahmed A. (2017). Expression of ribosomal proteins in normal and cancerous human prostate tissue. PLoS One.

[49] Destefanis F., Manara V., Bellosta P. (2020). Myc as a regulator of ribosome biogenesis and cell competition: a link to cancer. Int J Mol Sci.

[50] Monga J., Subramani D., Bharathan A., Ghosh J. (2020). Pharmacological and genetic targeting of 5-lipoxygenase interrupts c-Myc oncogenic signaling and kills enzalutamide-resistant prostate cancer cells svia apoptosis. Sci Rep.

[51] Coleman D.J., Gao L., King C.J., Schwartzman J., Urrutia J., Sehrawat A., Tayou J., Balter A., Burchard J., Chiotti K.E., Derrick D.S., Sun D., Xia Z., Heiser L.M., Alumkal J.J. (2019). BET bromodomain inhibition blocks the function of a critical AR-independent master regulator network in lethal prostate cancer. Oncogene.

[52] Fan L., Xu S., Zhang F., Cui X., Fazli L., Gleave M., Clark D.J., Yang A., Hussain A., Rassool F., Qi J. (2020). Histone demethylase JMJD1A promotes expression of DNA repair factors and radio-resistance of prostate cancer cells. Cell Death Dis.

[53] Santer F.R., Höschele P.P.S., Oh S.J., Erb H.H.H., Bouchal J., Cavarretta I.T., Parson W., Meyers D.J., Cole P.A., Culig Z. (2011). Inhibition of the acetyltransferases p300 and CBP reveals a targetable function for p300 in the survival and invasion pathways of prostate cancer cell lines. Mol Cancer Ther.

[54] Aggarwal R.R., Schweizer M.T., Nanus D.M., Pantuck A.J., Heath E.I., Campeau E., Attwell S., Norek K., Snyder M., Bauman L., Lakhotia S., Feng F.Y., Small E.J., Abida W., Alumkal J.J. (2020). A Phase Ib/IIa study of the Pan-BET bromodomain inhibitor ZEN-3694 in combination with enzalutamide in patients with metastatic castration-resistant prostate cancer. Clin Cancer Res.

