# Supplementary figures and images for: MYC-Mediated Ribosomal Gene Expression Sensitizes Enzalutamide-resistant Prostate Cancer Cells to EP300/CREBBP Inhibitors
Source: Am J Pathol. 2021 Mar 8;191(6):1094–107. doi: 10.1016/j.ajpath.2021.02.017 (PMC12178334; doi:10.1016/j.ajpath.2021.02.017)

**A**

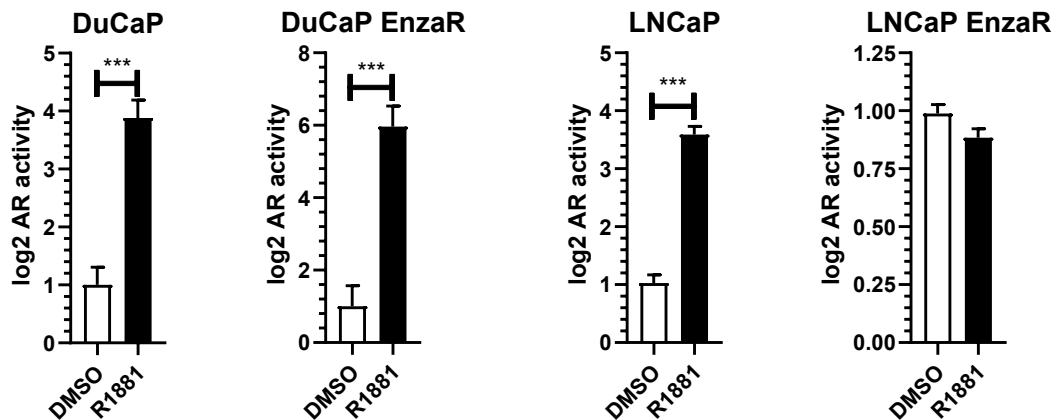

**B**

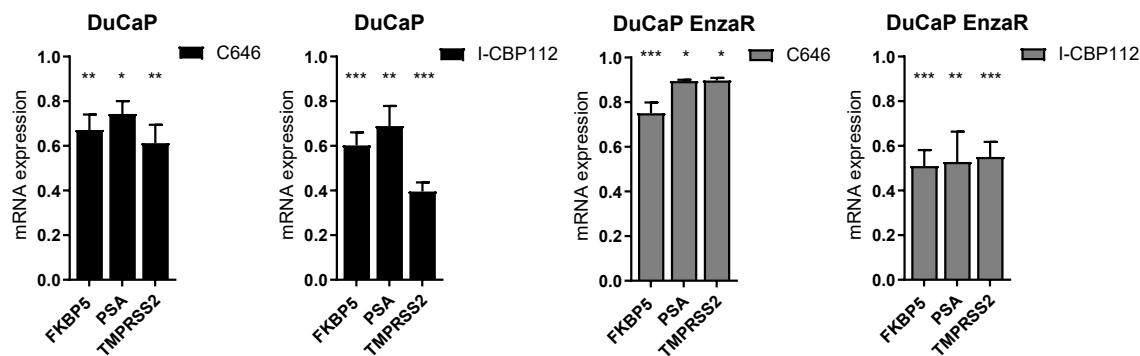

**C**

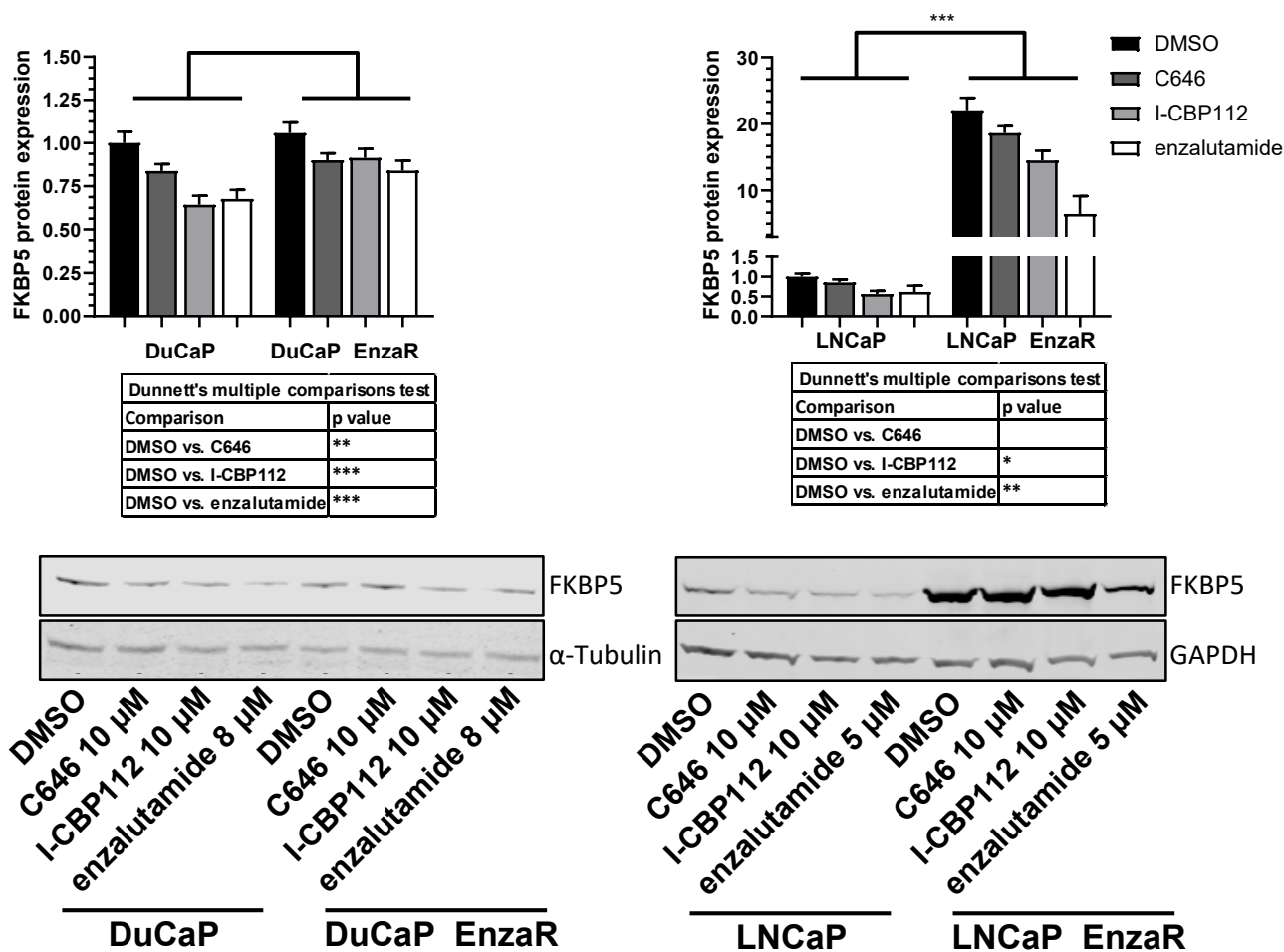

**Fig S1**

Supplement: Supplemental Figure S1 — A: Dual luciferase reporter assay for parental and resistant DuCaP and LNCaP cell lines treated with 10 nmol/L R1881 or dimethyl sulfoxide (DMSO) over 24 hours (n = 4). B: Quantitative real-time PCR of parental and enzalutamide-resistant DuCaP treated with DMSO, 10 μM C646, or 10 μM I-CBP112 for 24 hours in duplicates probed with primers specific for FKBP5, PSA, and TMPRSS2. Signals were normalized to the geometric mean of TBP, HPRT1, and HMBS expression (n = 3). Data are shown relative to DMSO-treated control. C: Representative Western blots for parental and resistant DuCaP and LNCaP cells treated with 10 μM C646, 10 μM I-CBP112, and 8 or 5 μM enzalutamide for 48 hours, probed for FKBP5 (n = 6). Numerical data were analyzed via one-way analysis of variance. ∗P < 0.05, ∗∗P < 0.01, ∗∗∗P < 0.001. [file mmc1.pdf]
